# Computing Leapfrog Regularization Paths with Applications to Large-Scale K-mer Logistic Regression

**DOI:** 10.1089/cmb.2020.0284

**Published:** 2021-06-14

**Authors:** Philipp Benner

**Affiliations:** Department of Computational Molecular Biology, Max Planck Institute for Molecular Genetics, Berlin, Germany.

**Keywords:** feature selection, $l_{1}$-regularization, LARS, orthogonal matching pursuit

## Abstract

**High-dimensional statistics deals with statistical inference when the number of parameters or features** *p* **exceeds the number of observations** *n* **(i.e.,** p≫n**). In this case, the parameter space must be constrained either by regularization or by selecting a small subset of** m≤n **features. Feature selection through** $l_{1}$**-regularization combines the benefits of both approaches and has proven to yield good results in practice. However, the functional relation between the regularization strength** λ **and the number of selected features** *m* **is difficult to determine. Hence, parameters are typically estimated for all possible regularization strengths** λ**. These so-called regularization paths can be expensive to compute and most solutions may not even be of interest to the problem at hand. As an alternative, an algorithm is proposed that determines the** $l_{1}$**-regularization strength** λ **iteratively for a fixed** *m***. The algorithm can be used to compute leapfrog regularization paths by subsequently increasing** *m***.**

## 1. Introduction

In statistics and machine learning, a common problem is to learn a model of the form

$$\mu_{\theta }\left(z\right)=\sigma \left(\theta_{1}f_{1}\left(z\right)+\ldots +\theta_{m}f_{m}\left(z\right)\right),$$

with coefficients $\theta =\left(\theta_{1},\ldots ,\theta_{m}\right)\in {ℛ}^{m}$ and where *f_j_* denotes the *j* th feature computed from the input data *z*. In statistics, σ is called *mean function*, whereas in machine learning it is commonly referred to as *activation function*. The model $\mu_{\theta }$ is typically estimated by minimizing a given loss function on a set of *n* observations.

For instance, in linear regression, the mean function is σ(x)=x and the parameters θ would be estimated by minimizing the sum of squared residuals, also called ordinary least squares (OLS). In the case of artificial neural networks, $\mu_{\theta }$ represents a single output neuron and *f_j_* denotes the *j*th output of the preceding layers of the network. The activation function σ is typically either a sigmoid function or some variant. Neural networks are estimated by minimizing, for instance, the mean squared or cross-entropy error.

Two fundamentally different ways can be found in the literature of how features *f_j_* are estimated. Some machine learning methods, such as neural networks, treat each *f_j_* as a parametric function, which is optimized jointly with the model parameters θ. In contrast, in statistics a fixed set of p>m features $\left\{x_{j}=f_{j}\left(z\right)\right\}$ is considered, from which a subset of size *m* is selected. Although the former is computationally more tractable, it often leads to nonconvex optimization problems. The focus of this study lies in the latter. In particular, it is assumed that the feature space is very large and that only a small subset of size *m* must be selected (p≫m). Ideally, the parameters of a statistical model are estimated for a given loss function $\omega :{ℛ}^{p}\to ℛ$ by minimizing ω(θ) subject to the constraint ${∥\theta ∥}_{0}=m$. However, this approach is generally not feasible since it requires to test all subsets of size *m*.

Several strategies exist to compute approximate solutions. For instance, matching pursuit (MP) (Tropp et al., 2007) is a greedy strategy that selects one feature at a time until the desired number of *m* features is reached. This algorithm was found to be overly greedy in practice (Efron et al., 2004) and may provide poor subset selections. A more powerful strategy is to consider the $l_{1}$ constraint ${∥\theta ∥}_{1}=\Lambda$ (Tibshirani, 1996). In practice, this leads to the equivalent unconstrained optimization problem

(P0) $$\hat{\theta }={\mathrm{argmin}}_{\theta }\omega \left(\theta \right)+\lambda {∥\theta ∥}_{1},$$

where the penalty strength λ≥0 is a parameter that controls the sparseness of θ and is often chosen so that ${∥\theta ∥}_{0}=m$. Although this approach often selects nearly optimal subsets, it comes with the difficulty of determining the penalty or regularization strength λ.

Unfortunately, there exists no known functional relation between *m* and λ. However, grid search can be used to compute solutions of the optimization problem P0 for a manually specified set of regularization strengths. More efficient algorithms, such as least angle regression (LARS) (Efron et al., 2004) and the homotopy (Osborne et al., 2000) algorithm, initially start with $\lambda =\lambda_{max}$ for which all coefficients are zero. These methods decrease λ and determine *breakpoints* $\lambda_{1}>\lambda_{2}>\ldots \lambda_{q}$ at which features either become active (nonzero coefficient) or inactive (zero coefficient). By interpolating between breakpoints a *regularization path* $\left\{\hat{\theta }\left(\lambda \right)|0\leq \lambda \leq \lambda_{max}\right\}$ is obtained, which contains solutions for all feasible regularization strengths.

Computing full regularization paths is costly for large feature spaces and complex models. In such cases, the regularization path is only computed up to a maximal $m_{q}\ll p$. Furthermore, a solution of the optimization problem P0 with *m* features will often behave very similarly to a solution with m+1 features. Therefore, the focus of this study is to present and test an algorithm that computes *leapfrog regularization paths* containing solutions $\hat{\theta }\left(\lambda_{k}\right)$ such that ${∥\hat{\theta }\left(\lambda_{k}\right)∥}_{0}=m_{k}$, where $m_{1}<m_{2}<\ldots <m_{q}$ is a given sequence of cardinalities. The main advantage of this algorithm is that *m_q_* can be chosen much larger than with existing regularization path algorithms, because it does not compute all solutions of the regularization path. Furthermore, the sequence of cardinalities $m_{1}<m_{2}<\ldots <m_{q}$ can be selected so that a clear difference in performance for *m_k_* and $m_{k+1}$ is observed.

## 2. Algorithmic Background

Many feature selection methods were first developed for linear regression and later extended to more complex models, such as generalized linear models (GLMs). Hence, linear regression may serve as a role model for introducing feature selection methods. Let $X\in {ℛ}^{n\times p}$ denote the covariates or data matrix with *n* observations $\left\{x_{i}\right\}\left(rows\right)$ and *p* features $\left\{f_{j}\right\}\left(columns\right)$. For linear regression, σ is the identity function so that $\mu_{\theta }\left(x_{i}\right)=x_{i}\theta$ and the objective function $\omega =\omega_{L}$ is the sum of squared residuals defined as


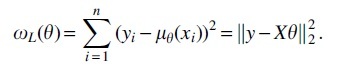


Furthermore, $y\in {ℛ}^{n}$ is a vector of *n* dependent variables, also called response, and ε=y−Xθ is referred to as residuals. If p≤n and *X* has full rank *p* then the coefficients θ can be estimated by OLS, which minimizes the sum of squared residuals $\omega_{L}\left(\theta \right)={∥\varepsilon ∥}_{2}^{2}={∥y-X\theta ∥}_{2}^{2}$. When p>n, parameters can be estimated by minimizing the $l_{1}$-penalized loss $L_{\lambda }\left(\theta \right)=\omega_{L}\left(\theta \right)+\lambda {∥\theta ∥}_{1}$, that is

(P1) $$\hat{\theta }={arg min}_{\theta }L_{\lambda }\left(\theta \right),$$

which is known as the Lasso (Tibshirani, 1996).

The following interpretation of OLS (Hastie et al., 2009) is essential for understanding many feature selection methods. In ${ℛ}^{n}$ the dot product Xθ is a point in the hyperplane *H* spanned by the columns $\left[f_{1},\ldots ,f_{p}\right]$ of *X* and *y* is a vector typically not contained in *H*. The point $X\hat{\theta }$ in *H* that minimizes the length of the residual vector ε is located directly below *y*, that is, it is the projection of *y* onto *H*. It follows that


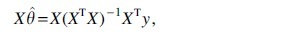


where 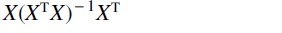 is a projection matrix. Therefore, the OLS solution 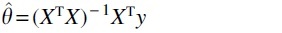 can be interpreted as the scalar projection of *y* onto *H*. If the covariates are appropriately normalized, that is, 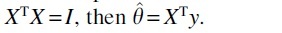 Most importantly, X^T^y can be interpreted as the correlation of *y* with *X*. The OLS solution satisfies 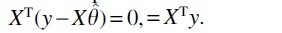, which shows that the residuals are uncorrelated with *X*. Many feature selection methods decrease the correlation 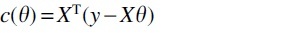 iteratively starting with c(0) until the residuals are orthogonal to *X*.

### 2.1. Matching pursuit

MP is a greedy feature selection algorithm (Tropp et al., 2007) that selects one feature at a time. Let the columns of *X* have unit length, that is, 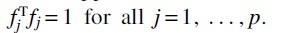 for all j=1,…,p. The first feature $j_{1}\in \left\{1,\ldots p\right\}$ is computed as 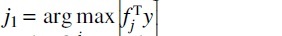. Hence, the algorithm selects the feature *j* with the largest scalar projection of *y* onto *f_j_*, or the feature that is most correlated with *y*.

All remaining features are selected accordingly. Let $\Omega =\left\{j_{1},\ldots ,j_{t}\right\}$ denote the set of active features and 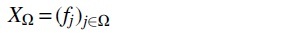 the covariate matrix restricted to Ω. Furthermore, let


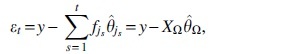


denote the residuals at iteration *t* with ${\hat{\theta }}_{\Omega }={\mathrm{argmin}}_{\theta }{∥y-X_{\Omega }\theta ∥}_{2}^{2}$. At iteration t+1 the algorithm computes

$$j_{t+1}={arg min}_{j}{∥\varepsilon_{t}-f_{j}{\hat{\theta }}_{j}∥}_{2}^{2}={arg max}_{j\notin \Omega }\left|f_{j}^{T}\varepsilon_{t}\right|,$$

that is, it selects that feature *j* for which the scalar projection of $\varepsilon_{t}$ onto *f_j_* is largest. Alternatively, 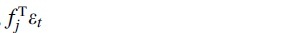 can be interpreted as the correlation of the *j*th feature with the residuals $\varepsilon_{t}$.

The MP algorithm has been applied to several other models, including logistic regression (Lozano et al., 2011), although the correlation 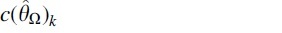 might be less informative.

### 2.2. LARS and homotopy algorithm

LARS is an iterative feature selection method for linear regression (Efron et al., 2004). The algorithm is related to MP, but it is less greedy and gives only as much weight to each feature as it deserves (Hastie et al., 2009). Another method is the homotopy algorithm (Osborne et al., 2000), which is identical to LARS except that it also allows features to be removed within an iteration. It is referred to as LARS with Lasso modification in the literature (Efron et al., 2004; Donoho and Tsaig, 2008). Both methods are introduced hereunder. *X* is typically assumed to be standardized so that the correlations c(θ) are not tainted by heterogeneous scaling.

The LARS and homotopy algorithms maintain a set of active features Ω⊂{1,…,p} all equally correlated with the residuals $y-X\hat{\theta }$ for the current estimate $\hat{\theta }$. In contrast, features not in Ω are less correlated with the residuals. Both algorithms initially set θ=0. In each subsequent iteration, the coefficients θ are updated so that the correlations of all features in Ω uniformly shrink toward zero until some other feature $j\prime \in \Omega^{c}$ is equally correlated with the residuals. For the next iteration, j′ is added to the active set Ω. The homotopy algorithm also removes a feature *j* from the active set Ω when the coefficient $\theta_{j}$ becomes zero. In this way, λ is incrementally reduced from its maximum value, where all features are inactive (Ω=⊘), to zero, where all coefficients are nonzero (Ω={1,2,…,p}). The values of λ at which a feature enters or leaves the active set Ω are referred to as breakpoints.

More specifically, the decrement in correlation at which a feature enters the active set is given by


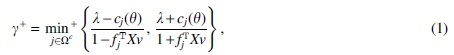


where min^+^ is the minimum over positive elements and $c_{j}\left(\theta \right)$ denotes the *j*th element of c(θ). For LARS without Lasso modification, the step size $\gamma^{*}$ equals $\gamma^{+}$; however, the homotopy algorithm also removes a feature *j* from the active set when for some γ

$$\theta_{j}+\gamma v_{j}=0,$$

so that $\gamma^{-}=\min_{j\in \Omega }\left\{-\theta_{j}∕v_{j}\right\}$. The subsequent breakpoint is given by $\gamma^{*}=\min\left\{\gamma^{+},\gamma^{-}\right\}$.

LARS with Lasso modification is equivalent to solving the Lasso problem (P1) for all regularization strengths λ≥0. A direct application of LARS or the homotopy algorithm to GLMs is not possible, because the path along which the correlations $c_{\Omega }\left(\theta \right)=\lambda 1$ uniformly decline is nonlinear. As a consequence, there is no analytical solution to the position of breakpoints. Nevertheless, a linear approximation for GLMs exists (Park and Hastie, 2007). The algorithm, however, requires several iterations until a breakpoint is reached.

## 3. Computing Leapfrog Regularization Paths

LARS and the homotopy algorithm compute full regularization paths $\left\{\hat{\theta }\left(\lambda \right)|0\leq \lambda \leq \lambda_{max}\right\}$ for all feasible regularization strengths λ. These algorithms start with $\lambda =\lambda_{max}$ and decrease the regularization strength until the unconstrained model is obtained. The full and exact regularization path is computed by linear interpolation between breakpoints. Extensions of LARS to GLMs (*LARS-GLM*) instead compute approximate regularization paths, because $\hat{\theta }\left(\lambda \right)$ is nonlinear between breakpoints and there exists no analytical expression to compute at what values of λ breakpoints occur.

For certain applications the full regularization path can be very costly to compute, especially when the number of features *p* is large. In such cases, the algorithms can be stopped when a maximum number of features *m_q_* is reached. Nevertheless, because LARS-GLM successively decreases the regularization strength λ, it might take very long until a desired number of features *m* is reached. Furthermore, estimated models with *m* and m+1 selected features might behave very similarly, for instance, in terms of classification performance. For such situations, an algorithm is introduced that computes for an a priori defined sequence $m_{1}<m_{2}<\ldots <m_{q}$ the estimates $\hat{\theta }\left(\lambda_{k}\right)$ such that ${∥\hat{\theta }\left(\lambda_{k}\right)∥}_{0}=m_{k}$. The set of estimates ${\left\{\hat{\theta }\left(\lambda_{k}\right)|{∥\hat{\theta }\left(\lambda_{k}\right)∥}_{0}=m_{k}\right\}}_{k=1}^{q}$ is called a leapfrog regularization path. The algorithm can be derived from proximal gradient descent (Boyd et al., 2004) or from a modification of LARS-GLM.

Let ω denote the objective function and $\omega_{j}^{\prime }\left(\hat{\theta }\right)=\frac{\partial }{\partial \theta_{j}}\omega \left(\theta \right){|}_{\theta =\hat{\theta }}$ the *j*th partial derivative at the current estimate $\hat{\theta }$. Given a fixed $m\in \left\{m_{1},m_{2},\ldots ,m_{q}\right\}$ the following update rules are iterated until convergence:


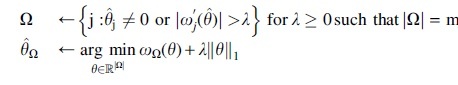


The algorithm has converged when Ω is identical between two successive iterations. The first update step computes a new active set Ω. All features with nonzero coefficients remain in the active set. Additional features are added based on the absolute value of the partial derivatives. An estimate of the optimal λ parameter is obtained during this step. In the second step, the coefficients of the active set are re-estimated, all other coefficients remain zero. The re-estimation is computationally cheap if m≪p, because the gradient descent method operates only on *m* selected features. Once the algorithm has converged for a fixed $m=m_{k}$, the procedure is repeated for $m=m_{k+1}$ (see Algorithm 1).

**Table:** 

**Algorithm 1** Calculate leapfrog regularization path ${\left\{\hat{\theta }\left(\lambda_{k}\right)|{∥\hat{\theta }\left(\lambda_{k}\right)∥}_{0}=m_{k}\right\}}_{k=1}^{q}$
1: **procedure**LeapfrogPath$\left(m_{1}<m_{2}<\ldots <m_{q}\right)$
2: r←⊘
3: $\hat{\theta }\leftarrow 0\in {ℛ}^{p}$
4: **for**k=1,…,q**do**
5: **repeat**

${\hat{\theta }}_{\Omega }\leftarrow {arg min}_{\theta \in {ℛ}^{\left|\Omega \right|}}\omega_{\Omega }\left(\theta \right)+\lambda {∥\theta ∥}_{1}$
6: **until**Ω remains unchanged
7: $r\leftarrow r\cup \left\{{\hat{\theta }}_{\Omega }\right\}$
8: **end for**
9: **return***r*
10: **end procedure**

Algorithm 1 relies on $l_{1}$-regularization for feature selection. Compared with MP it is much less greedy, because it does not simply select one feature per iteration (Hastie et al., 2009). Instead, the active set is refined until a solution to optimization problem (P0) is found. The algorithm is more similar to LARS-GLM, but it uses a different method for determining the regularization strength λ. Whereas LARS estimates the position of subsequent breakpoints, Algorithm 1 iteratively updates λ until a given number of *m* features is selected.

Despite extremely high-dimensional feature space, the estimation of parameters is relatively cheap when m≪p, because the gradient descent method operates only on a small subset of *m* features and not the full feature space of size *p*. In other words, estimating the coefficients of the logistic regression with a gradient method takes O(nmT) steps, where *T* is the number of iterations of the gradient descent method. This is much faster than estimating the parameters on the full feature space, which would take O(npT) steps.

### 3.1. Derivation from proximal gradient descent

The optimization problem P0 is commonly solved using proximal gradient descent (Boyd et al., 2004). In general, proximal gradient descent can be used to compute minimizers of functions f(x)=g(x)+h(x), where *g* is convex differentiable and *h* is convex but not differentiable. The update equation is given by

$$x\leftarrow prox_{h}\left(x-\gamma \nabla g\left(x\right)\right),$$

$$whereprox_{h}\left(x\right)={arg min}_{x\prime }\frac{1}{2\gamma }{∥x\prime -x∥}_{2}^{2}+h\left(x\prime \right)$$

and γ is the step size. The proximal operator $prox_{h}$ can be solved analytically for $h\left(x\right)={∥x∥}_{1}$. Note that the equation inside the proximal operator is a simple gradient descent step applied to g..

For the optimization problem P0 the following update equations are obtained:

ϑ←θ−γ∇ω(θ)

$$\theta \leftarrow {arg min}_{\theta \prime }\frac{1}{2\gamma }{∥\theta \prime -\vartheta ∥}_{2}^{2}+{∥\theta \prime ∥}_{1}=sign\left(\vartheta \right)\max\left\{|\vartheta |-\gamma \lambda ,0\right\},$$

where ϑ is the result after the gradient descent step and before applying the proximal operator. Assume that for a feature *j* the coefficient $\theta_{j}$ is zero. The coefficient remains zero during subsequent update steps unless the gradient update is large enough, because of the application of the proximal operator. More specifically, for any step size γ>0, a coefficient $\theta_{j}$ remains zero unless $|\omega_{j}^{\prime }\left(\hat{\theta }\right)|>\lambda$.

### 3.2. Derivation from LARS

In each iteration, LARS either removes a feature from the active set or selects a new one. The focus here is on the selection of new features, that is, Equation (1). By assuming that $f_{j}^{T}Xv=0$, a much simplified step size


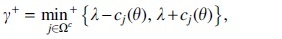


is obtained, which shows that $\lambda -\gamma^{+}=\max_{j\in \Omega^{c}}|c_{j}\left(\theta \right)|$ and remember that for linear and logistic regression $c_{j}\left(\theta \right)=\omega \prime_{j}\left(\theta \right)$. Hence, *m* features can be selected by setting λ equal to the *m*th largest absolute correlation or partial derivative.

## 4. Applications to Large-Scale Logistic Regression

Algorithm 1 can be easily applied to GLMs, such as logistic regression, as demonstrated in the following. The closest method in the literature is LARS-GLM and the *GNU-R* library *glmpath* is used for comparing it with Algorithm 1. Decisive for the computational cost is the number of *steps* required for computing a regularization path. A step here refers to the estimation of parameters for a fixed regularization strength λ, that is, solving optimization problem P0. For Algorithm 1 this corresponds to one evaluation of the two update equations. In addition, the number of passes of the gradient descent method through the entire data set (*epochs*) is informative. However, because different gradient descent methods are used for Algorithm 1 and LARS-GLM, a direct comparison in terms of epochs is not possible.

Enhancers are regulatory elements in the genome that control the cell type-specific expression of genes. The DNA sequence of an enhancer is predictive of the cell types in which it actively controls gene expression (Lee et al., 2011; Hashimoto et al., 2016; Kelley et al., 2016; Yang et al., 2017). Training and test data sets were compiled from DNA sequences of 56,880 enhancers active in embryonic mice at day 15.5 in brain (positive set) and nonbrain tissues (negative set). Enhancers were detected using ModHMM (Benner and Vingron, 2020) and the data are available online (Benner, 2020). For each enhancer sequence, the *K*-mer content is determined. *K*-mers are short DNA subsequences of length *K*. In this example, *K*-mers of variable length are considered. The resulting data matrix $X=\left(x_{ij}\right)$ has *n* rows (observations) and *p* columns (features), where $x_{ij}$ is the number of occurrences of the *j*th *K*-mer in the *i*th enhancer sequence. A logistic regression model is used to discriminate between brain and nonbrain enhancers. The average negative log-likelihood (*average loss*) is given by


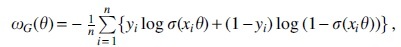


where σ is the sigmoid function and $y\in {\left\{0,1\right\}}^{n}$ is a vector of *n* class labels. Analogously to linear regression, when p>n, parameters are estimated by minimizing the $l_{1}$-penalized average logistic loss $G_{\lambda }\left(\theta \right)=\omega_{G}\left(\theta \right)+\lambda ∥\theta ∥$ (*penalized average loss*), that is

$$\hat{\theta }={\mathrm{argmin}}_{\theta }G_{\lambda }\left(\theta \right).$$

For logistic regression the objective function $G_{\lambda }$ is convex and the optimum $\hat{\theta }$ must satisfy 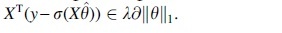. SAGA (Defazio et al., 2014) is used as gradient descent method to compute solutions of this optimization problem. To guarantee stable convergence of the gradient descent method, the data are normalized to unit variance.

**FIG. 1.**
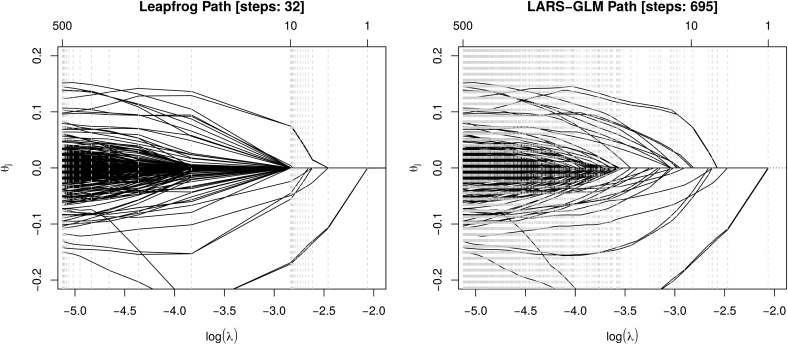
Comparison of regularization paths. Leapfrog regularization path (left) with m∈{1,10,500} and LARS-GLM regularization path (right). Vertical dashed gray lines show steps of the path algorithms, that is, λ values for which parameters $\hat{\theta }$ were estimated

In a first example, *K*-mers of length 2−7 are used, that is, K∈{2,3,…,7}. This leads to a feature space of size p=21,840, where only *K*-mers that actually occur in the data set are considered. Figure 1 shows the leapfrog regularization path computed with Algorithm 1 for m∈{1,10,500}. In addition, a regularization path computed with LARS-GLM is shown and the algorithm was stopped when it reached 500 features. The example shows that the computation of the leapfrog path requires much fewer steps, however, at the expense of not evaluating the full path up to 500 features. Nevertheless, for larger feature spaces and larger *m* the LARS-GLM algorithm quickly becomes computationally too expensive.

**FIG. 2.**
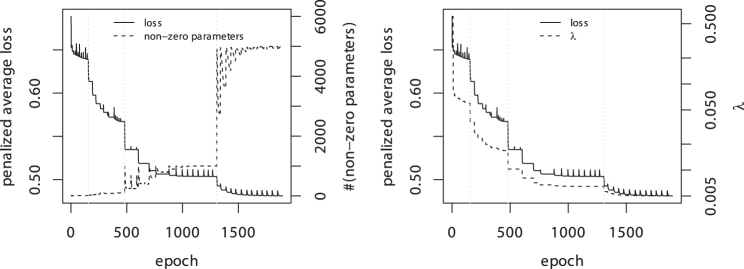
Estimation of a leapfrog regularization path with m∈{10,100,1000,5000}. The vertical lines show the epoch at which *m* is increased. An epoch is one pass of the gradient descent method through the entire data. For a fixed penalty strength λ, the gradient descent method is iterated until convergence. If the number of nonzero parameters does not match the target *m*, λ is updated and the parameters are re-estimated.

In a second example, the set of *K*-mers is extended to lengths of 2−12, that is, K∈{2,3,…,12}, which results in a feature space of size p=16,876,344. Figure 2 shows the estimation of parameters for a leapfrog regularization path with Algorithm 1 and targets m∈{10,100,1000,5000}. The algorithm successively decreases the penalty strength λ until a target is reached. After only 1500 epochs, corresponding to ∼50 steps of Algorithm 1, the leapfrog regularization path is computed. In contrast, extracting the 5000 most important *K*-mers with the LARS-GLM algorithm is computationally not feasible.

### 4.1. Bias-variance trade-off

In statistics and machine learning one is often confronted with the task of choosing a model family ℍ that captures the underlying structure of the observed data, but also generalizes well to new observations. The bias-variance trade-off suggests that both cannot be accomplished with arbitrary precision. When the capacity of ℍ is too small, the estimated models has poor performance on the observed data as well as on new observations. The underfitting of the estimated models is caused by a strong (inductive) *bias* of ℍ. As the capacity of ℍ increases, the model is capable of extracting more complex patterns from the training data, but the *variance* of parameter estimates across samples increases. If the capacity of ℍ is too large it causes overfitting, that is, the estimated models will perform very well on the training data but generalize poorly. A model family that generalizes well must minimize both sources of error, the bias and the variance.

For instance, in the case of linear or logistic regression, the capacity of ℍ can be increased by using more features (i.e., increasing *p*). Regularization, in contrast, constrains the parameter space and, therefore, reduces the capacity of ℍ (Hastie et al., 2009). In practice, it is often observed that models generalize best if regularization is used in combination with very large *p* (Bühlmann and Van De Geer, 2011).

**FIG. 3.**
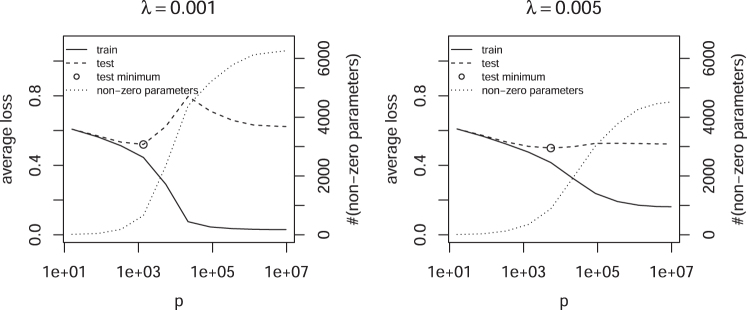
Average loss as a function of *p* and fixed penalty λ.

In this section, the effect of regularization and the size of the feature space *p* on the capacity of ℍ is studied. A balanced set of *n* = 10,000 brain and nonbrain enhancers is used. The size of the feature space *p* is controlled by the maximal length of *K*-mers. Figure 3 shows the training and test error for a fixed penalty λ and increasing *p*. Whereas the training error decreases monotonically, the test error starts to decrease after it reaches a maximum. This double descent has been observed before (Vallet et al., 1989; Opper et al., 1990; Duin, 2000; Belkin et al., 2019) and is theoretically expected (Cheema and Sugiyama, 2020). It shows that the capacity of ℍ is the result of a complex interplay between the size of the feature space *p* and the regularization strength λ.

**FIG. 4.**
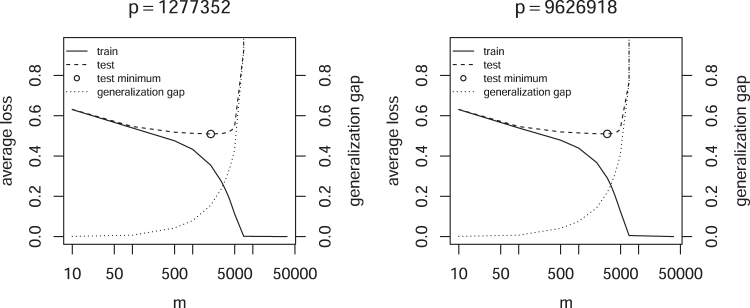
Average loss as a function of *m* and fixed *p*.

The dependency between λ and *p* makes it difficult in practice to find optimal values for both parameters, especially when double descents are possible, because an extensive search is required to eliminate the possibility of being in a local optimum. Instead of adding more parameters by increasing *p*, Algorithm 1 is utilized to control the number of active features *m* for a fixed *p*. Figure 4 shows that in this case no double descent is observed, suggesting a monotonic relation between *m* and the capacity of ℍ, as expected from the bias-variance trade-off. Furthermore, the test error increases rapidly as the interpolation threshold is crossed, that is, the point where the model is able to perfectly discriminate between the positive and negative class.

**FIG. 5.**
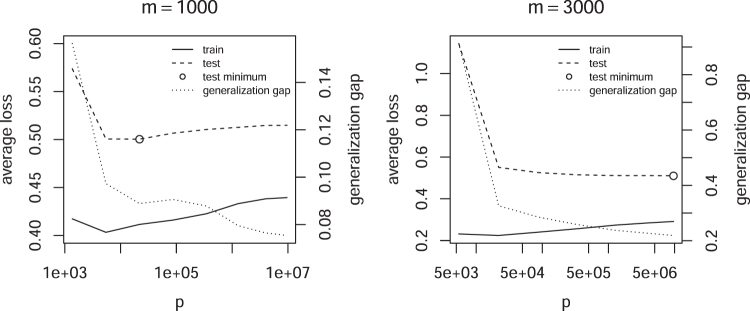
Average loss as a function of *p* and fixed *m*.

Figure 5 shows the converse case, the average loss for fixed *m* but variable *p*. Also in this case no double descent of the test error is observed. However, the training error increases with *p* after reaching a minimum, because the regularization strength must increase to keep *m* fixed. The generalization gap, that is, the difference between test and training loss, steadily decreases, showing that the capacity of ℍ seems to decline.

## 5. Conclusion

Several iterative feature selection methods were discussed in this study. MP is among the simplest methods, but can lead to poor subset selection because of its overly greedy strategy (Hastie et al., 2009). More advanced methods, such as LARS and the homotopy algorithm, often yield better results in practice. The estimates of LARS with Lasso modification are equivalent to solving the Lasso problem (P1) for all regularization strengths λ. Extensions to GLMs are more expensive to compute, because there exists no analytical solution to the λ values at which the active set of features changes. As a result, many more iterations are required to compute a full regularization path. In practice, one is often not interested in the full solution, because estimates with *m* and m+1 features provide very similar predictions.

Algorithm 1 instead computes for an a priori defined sequence $m_{1}<m_{2}<\ldots <m_{q}$ a leapfrog regularization path ${\left\{\hat{\theta }\left(\lambda_{k}\right)|{∥\hat{\theta }\left(\lambda_{k}\right)∥}_{0}=m_{k}\right\}}_{k=1}^{q}$. This algorithm can be derived from proximal gradient descent or a simple modification of LARS for GLMs. It is very effective for high-dimensional problems where only a small subset of features is required for making accurate predictions. It is less greedy than MP, because it does not simply select one feature per iteration. As opposed to LARS, which computes subsequent breakpoints, Algorithm 1 iteratively refines the regularization strength λ until a given number of *m* features is selected.

On a data set of DNA sequences the algorithm very efficiently computes the leapfrog regularization path despite the high-dimensional feature space, where LARS-GLM is computationally too expensive. In addition, the number of selected features *m* allows a good control of model complexity. An implementation of the algorithm is available at: https://github.com/pbenner/kmerLr
